# The Delta Neutrophil Index Predicts the Development of In-hospital Hypotension in Initially Stable Patients with Pyogenic Liver Abscess

**DOI:** 10.1038/s41598-019-48588-1

**Published:** 2019-08-20

**Authors:** Taeyoung Kong, Yoo Seok Park, Hye Sun Lee, Sinae Kim, Jong Wook Lee, Je Sung You, Hyun Soo Chung, Incheol Park, Sung Phil Chung

**Affiliations:** 1 Department of Emergency Medicine, Yonsei University College of Medicine, Seoul, Republic of Korea; 2 Department of Research Affairs, Biostatistics Collaboration Unit, Yonsei University College of Medicine, Seoul, Republic of Korea; 3 Department of Laboratory Medicine, Konyang University Hospital, Daejeon, Republic of Korea

**Keywords:** Prognostic markers, Liver diseases

## Abstract

Prompt diagnosis and timely treatment are important for reducing morbidity and mortality from pyogenic liver abscess (PLA). The purpose of this study was to investigate the importance of the delta neutrophil index (DNI) reflecting the fraction of immature granulocytes as a predictor of the development of in-hospital hypotension in initially stable patients with PLA. We retrospectively identified 308 consecutive patients (>18 years) who were hemodynamically stable at presentation and diagnosed with PLA in the emergency department (ED) between January 2011 and September 2017. The outcome of interest was in-hospital hypotension 1–24 hours after admission to the ED. A high DNI at ED admission was an independent predictor of the development of in-hospital hypotension in initially stable patients with PLA (odds ratio [OR]: 1.44, 95.0% confidence interval [CI]: 1.06–1.95; *P* = 0.02). A DNI > 3.3% was associated with in-hospital hypotension at ED admission (OR: 5.37, 95.0% CI: 2.91–9.92; P < 0.001). The development of in-hospital hypotension was associated with an increased risk of 30-day mortality (HR: 8.55, 95.0% CI: 2.57–28.4; *P* < 0.001). A high DNI independently predicts the development of in-hospital hypotension in initially stable patients with PLA. In-hospital hypotension is associated with an increased risk of 30-day mortality.

## Introduction

Although pyogenic liver abscess (PLA) is uncommon, its incidence is increasing worldwide^1^. PLA is more prevalent in Asian countries than in Western countries^2^. Recent significant increases in incidence in the United States may be explained by the increasing aging population, diabetes, hepatobiliary disease, instrumental usage of the biliary tract, and liver transplantation^3^.

In the 1930s, PLA was considered a potentially life-threatening condition, with mortality rates of up to 77.0%^4^. The current mortality rate of PLA has markedly reduced to 6.0–14.0%^5^. Despite advances in diagnostic and therapeutic techniques, PLA remains a critical illness with significant morbidity and mortality rates^5^. As early and appropriate applications of intravenous antibiotics and catheter drainage are the main therapies for PLA, prompt diagnosis and timely treatment are most important for PLA^4,6^. Although these patients have stable hemodynamics in the early stages of PLA, delays in appropriate diagnosis and treatment can lead to the rapid development of septic shock^2^. Septic shock is significantly associated with tissue hypoperfusion, increased development of organ failure, and eventual mortality^2,7^. Therefore, prompt and accurate risk stratification for the development of in-hospital hypotension in initially stable patients with PLA plays a key role in improving outcomes^2^. However, it is difficult to predict the development of in-hospital hypotension in patients with PLA based on several known prognostic indicators.

Recent technological advances enable us to determine the delta neutrophil index (DNI), reflecting the fraction of circulating immature granulocytes, using a specific automated blood cell analyzer^8–11^. The DNI, measured by an automated blood cell analyzer, is strongly associated with manually counted immature granulocytes^12^. The DNI overcomes the disadvantages of manually counting immature granulocytes and can aid in predicting infection severity in early stages^13^. An increase in circulating immature granulocytes, as well as a leftward granulocytic shift, are well-known to be associated with infection and systemic inflammation^8–11,14,15^. Several studies^12,13^ have reported that a high DNI reflecting an increase in the fraction of immature granulocytes is significantly associated with a positive blood culture test result, the development of septic shock, disseminated intravascular coagulation, and mortality in critically ill patients with suspected sepsis.

To the best of our knowledge, few studies have investigated the usefulness of biomarkers in predicting the development of in-hospital hypotension, and this is the first study to evaluate the relationship between DNI and the development of in-hospital hypotension in initially hemodynamically stable patients with PLA. We investigate the importance of DNI as a predictor of the development of in-hospital hypotension in initially stable patients with PLA.

## Results

### Study population, clinical evaluation, and treatment

**Figure 1:**
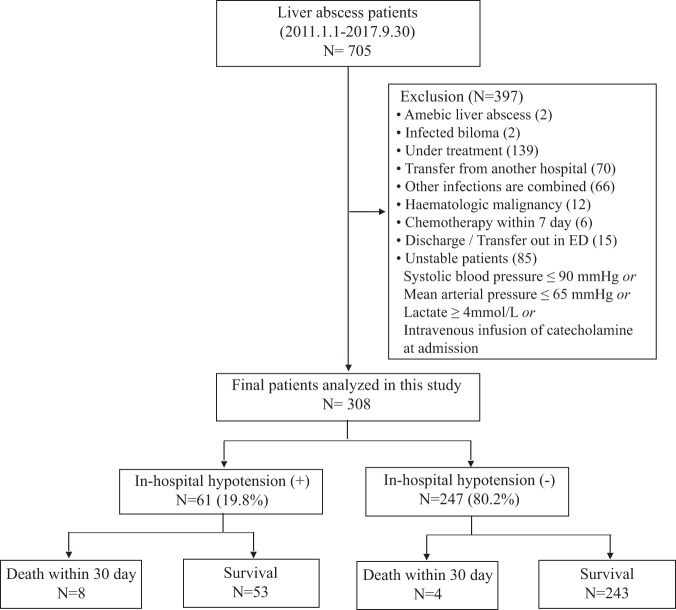
Flow chart of patient enrolment.

The inclusion and clinical outcomes of the initially stable patients with PLA (*n* = 308; 63[55–71] years) are shown in Fig. 1. Sixty-one patients (19.8%) developed in-hospital hypotension. The 30-day mortality rate was 3.9%. Twelve of the 308 patients (3.9%) died within 30 days after ED admission because of septic shock after PAL (n = 8), hepatic failure due to hepatocellular carcinoma (n = 1), aggravation of cholangiocarcinoma (n = 1) and pancreatic cancer (n = 1), and unexpected cardiac arrest during endoscopy (n = 1). Patients were initially treated with empirical intravenous broad-spectrum antibiotics. Percutaneous drainage was performed in 204 patients (66.2%). Surgical intervention was performed in 6 patients (1.9%) (Table 1). Isolated bacterial characteristics are shown in Table 2. The present study identified *Klebsiella pneumoniae* as the most common pathogen in blood (n = 74, 24%) and pus (n = 104, 49.5%) cultures. Among the patients with *K*. *pneumoniae* in the blood, 20 of 74 had malignancy of the gastrointestinal tract. These malignancies included hepatocellular carcinoma (n = 6), pancreatic cancer (n = 4), stomach cancer (n = 3), duodenal cancer (n = 1), ampulla of vater cancer (n = 1), cholangiocarcinoma (n = 4), and colon cancer (n = 1). Furthermore, 24 of the 104 patients with *K*. *pneumoniae* in pus had malignancy of the gastrointestinal tract. By type, they had hepatocellular carcinoma (n = 5), gallbladder cancer (n = 4), pancreatic cancer (n = 2), stomach cancer (n = 2), duodenal cancer (n = 1), ampulla of vater cancer (n = 2), cholangiocarcinoma (n = 5), rectal cancer (n = 1), and colon cancer (n = 2).

**Table 1:** Clinical characteristics of patients according to the development of in-hospital hypotension.

Variables	Total N = 308 (100%)	Development of in-hospital hypotension		
		No N = 247 (80.2%)	Yes N = 61 (19.8%)	*P*
Age (years)	63 [55–71]	62 [54–71]	66 [56–73]	0.09
Male gender [n (%)]	192 (62.34)	155 (62.75)	37 (60.66)	0.76
qSOFA score (point)	0 [0–1]	0 [0–1]	1 [0–1]	<0.01*
**Initial vital sign**				
MBP (mmHg)	85 [77–96]	87 [78–98]	78 [73–87]	<0.01*
Heart rate (bpm)	96 [85–108]	94 [83–106]	105 [95–120]	<0.01*
Body temperature (°C)	37.7 [36.9–38.7]	37.6 [36.8–38.5]	38.3 [37.1–39.2]	0.01*
**Radiology features**				
Size of abscess (cm)	5 [3.3–6.8]	5 [3.3–6.8]	5 [3.4–6.5]	0.91
Multiple lesions [n (%)]	94 (30.5)	74 (30)	20 (32.8)	0.67
Bilobar involvement [n (%)]	47 (15.3)	35 (14.2)	12 (19.7)	0.29
**Treatment modality** [n (%)]				0.15
Antibiotic only	98 (31.8)	81 (32.8)	17 (27.9)	
Antibiotics + Percutaneous drainage	204 (66.2)	163 (66)	41 (67.2)	
Antibiotics + Surgical treatment	6 (2)	3 (1.2)	3 (4.9)	
**Timing of treatment**				
Door to antibiotic time (hour)	3.98 [2.88–5.13]	3.98 [2.83–5.2]	3.93 [3.15–5.08]	0.88
Door to intervention time (hour)	15.84 [4.97–38.24]	15.72 [4.83–27.82]	17.02 [5.3–80.65]	0.12
**Comorbidity** [n (%)]				
Hypertension	120 (38.96)	96 (38.87)	24 (39.34)	0.95
Diabetes mellitus	84 (27.27)	67 (27.13)	17 (27.87)	0.91
Chronic kidney disease	7 (2.27)	4 (1.62)	3 (4.92)	0.14
Malignancy	121 (39.29)	92(37.25)	29 (47.54)	0.14
Liver cirrhosis	9 (2.92)	5 (2.02)	4 (6.56)	0.08
Cardiovascular disease	29([9.42)	22 (8.91)	7(11.48)	0.54
**Laboratory data**				
WBC count (10^3/μL)	11.11 [7.66–15.23]	11.36 [8.32–15.06]	8.96 [6.04–15.35]	0.11
Hemoglobin (g/dL)	12.15 [10.7–13.4]	12.2 [10.8–13.5]	11.7 [10.5–13.2]	0.37
Platelet count (10^3/μL)	178 [124–272]	192 [136–301]	132 [88–198]	<0.01*
Neutrophil ratio (%)	85.4 [79.9–89.5]	83.95 [79.05–88.2]	89.5 [85.4–93]	<0.01*
Prothrombin ratio (%)	80 [69–93]	80 [70–95]	79 [66–89]	0.2
Creatinine (mg/dL)	0.85 [0.65–1.08]	0.8 [0.63–1.01]	1.02 [0.81–1.5]	<0.01*
Albumin (g/dL)	3.2 [2.8–3.6]	3.2 [2.8–3.6]	2.9 [2.7–3.4]	0.01*
ALT (IU/L)	40 [25–74]	38 [24–66]	56 [27–96]	0.02*
Total bilirubin (mg/dL)	1.1 [0.7–1.7]	0.9 [0.6–1.6]	1.3 [1–2.4]	<0.01*
C reactive protein (mg/L)	169 [95–234]	165 [90–228]	197 [134–266]	0.02*
Lactate (mmol/L)	1.75 [1.1–2.8]	1.3 [1–2]	2.6 [1.6–4.8]	<0.01*
Bacteremia [n (%)]	111 (36)	64 (25.9)	47 (77.1)	<0.01*
DNI^a^	0.9 [−0.29–1.61]	0.74 [−0.69–1.44]	1.79 [0.99–2.73]	<0.01*

*P < 0.05, Data are expressed in median [IQR] and n (%), ^a^Variable was logarithmically transformed before analyses.

Abbreviations: qSOFA, quick sequential organ failure assessment; MBP, mean blood pressure; WBC, white blood cell; AST, aspartate aminotransferase; ALT, alanine aminotransferase; DNI, delta neutrophil index.

**Table 2:** Bacterial isolates from 308 patients with pyogenic liver abscess.

Pathogen	Blood (N = 308)	Pus (N = 210)
Klebsiella pneumoniae	74 (24%)	104 (49.5%)
Escherichia coli	9 (2.9%)	19 (9.0%)
Streptococcus species	5 (1.6%)	11 (5.2%)
Staphylococcus species	0 (0%)	2 (1.0%)
Enterococcus species	2 (0.6%)	5 (2.4%)
Enterobacter species	5 (1.6%)	4 (1.9%)
Clostridium species.	2 (0.6%)	2 (1.0%)
Anaerobes	6 (1.9%)	5 (2.4%)
Others	8 (2.6%)	2 (1.0%)
No growth	197 (64%)	56 (26.7%)

### DNI as a Predictor of in-hospital hypotension in initially stable patients with PLA

**Figure 2:**
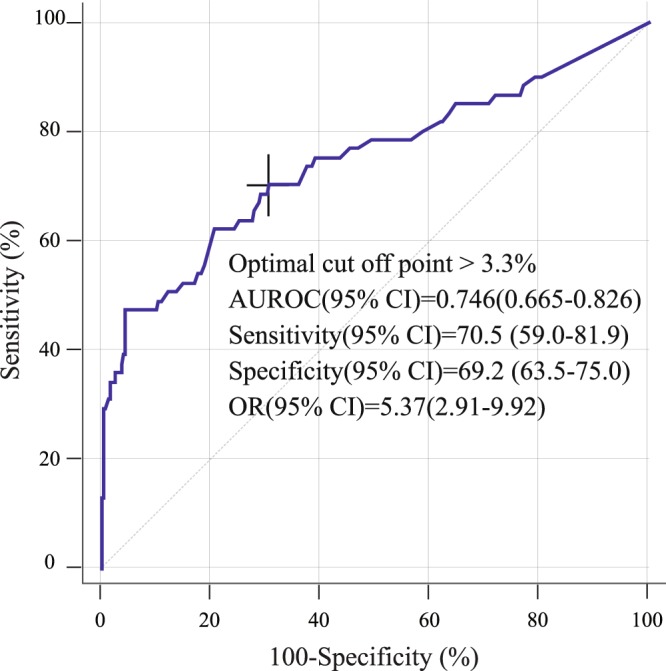
ROC curves of the DNI for predicting the development of in-hospital hypotension. DNI, delta neutrophil index; ROC, receiver operating characteristic.

**Figure 3:**
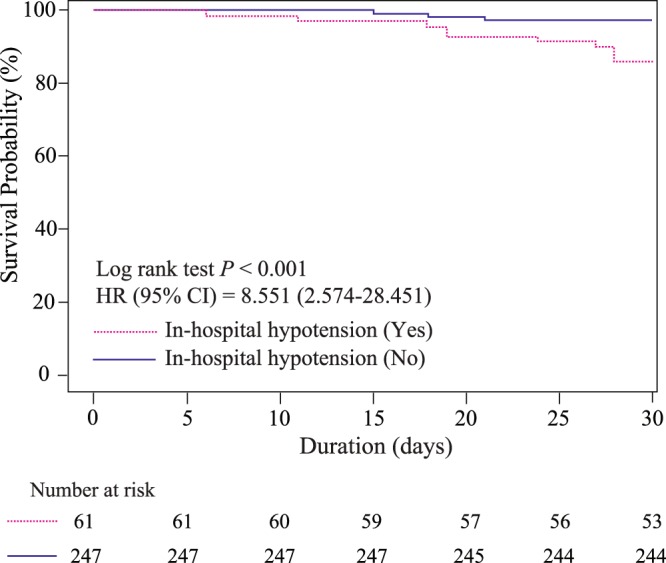
The development of in-hospital hypotension as a predictor of 30-day mortality.

Distribution of delta neutrophil index (DNI) according to development of in-hospital hypotension are shown in Supplement 1. The DNI vale was higher in patients with in-hospital hypotension than in those without in-hospital hypotension (Table 1). Univariable logistic regression analysis revealed that the DNI was significantly different at ED admission between those who did and did not develop in-hospital hypotension (Supplement 2). In the multivariable logistic regression analysis, a high DNI at ED admission was also an independent predictor of the development of in-hospital hypotension in initially stable patients with PLA (odds ratio [OR]: 1.44, 95.0% confidence interval [CI]: 1.06–1.95; *P* = 0.02) (Table 3). The AUROC for predicting in-hospital hypotension using the DNI at ED admission in initially stable patients with PLA was 0.746 (*P* < 0.001) (Fig. 2). Using Youden’s index, the optimal cutoff value for the DNI at ED admission was 3.3% (sensitivity, 70.5 [59–81.9]; specificity, 69.2 [63.5–75]). A DNI > 3.3% was significantly associated with in-hospital hypotension at ED admission (OR: 5.37, 95.0% CI: 2.91–9.92; *P* < 0.001) (Fig. 2). Kaplan-Meier curves showed a significant association between in-hospital hypotension and 30-day mortality (Hazard ratio (HR): 8.55, 95.0% CI: 2.57–28.4; *P* < 0.001) in initially stable patients with PLA (Fig. 3).

**Table 3:** Multivariable logistic regression analysis of predictors for the development of in-hospital hypotension.

Variable	Multivariable logistic regression	
	OR (95% CI)	*P*
qSOFA score	2.453 (1.451–4.147)	0.004*
Heart rate (per 1bpm)	1.030 (1.011–1.050)	0.004*
Body temperature (per 1 °C)	1.314 (0.939–1.837)	0.075
Platelet count (per 10^3/μL)	0.996 (0.993–0.999)	0.012*
Neutrophil ratio (per 1%)	0.990 (0.946–1.036)	0.657
Prothrombin time (per 1%)	0.993 (0.972–1.016)	0.555
Albumin (per 1 g/dL)	0.656 (0.326–1.319)	0.237
ALT (per 1IU/L)	1.003 (0.999–1.007)	0.186
Total bilirubin (per 1 mg/dL)	1.009 (0.906–1.125)	0.866
C reactive protein (per 1 mg/L)	1.001 (0.997–1.005)	0.584
DNI^a^ (per 1%)	1.437 (1.058–1.953)	0.020*

*P < 0.05, ^a^Variable was logarithmically transformed before analyses.

Abbreviations: ALT, alanine aminotransferase; DNI, delta neutrophil index; qSOFA, quick sequential organ failure assessment.

### Comparison of the DNI and conventional clinical markers as predictors of in-hospital hypotension in initially stable patients with PLA

**Figure 4:**
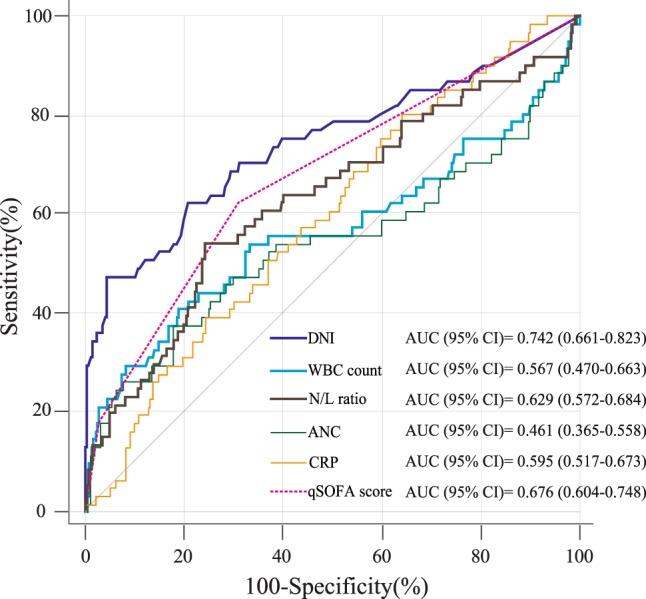
Comparison of the ROC curves of the DNI and other laboratory markers for predicting the development of in-hospital hypotension. Abbreviations: DNI, delta neutrophil index; ROC, receiver operating characteristic; WBC, white blood cell; N/L, neutrophil/lymphocyte; ANC, absolute neutrophil count; CRP, C-reactive protein; qSOFA, Quick Sequential Organ Failure Assessment.

To predict the development of in-hospital hypotension, comparisons of the ROC curves showed that the AUROC for the DNI at ED admission was significantly superior to that of other markers (C-reactive protein [CRP], white blood cell count [WBC], neutrophil-to-lymphocyte ratio [NLR], and absolute neutrophil count [ANC]). Moreover, the DNI value was not significantly inferior to those of other markers, including qSOFA scores at ED admission (Fig. 4 and Supplement 3).

### Prognostic value of the DNI in combination with conventional risk factors

Integrated discrimination improvement (IDI) and net reclassification improvement (NRI) are proposed indicators for identifying improvement of reclassification in a nested model, showing how predictive power improves when DNI is added to traditional risk factors. The addition of DNI yielded a significantly positive IDI and NRI for the prediction of in-hospital hypotension. However, no significant improvement was observed in the AUC when DNI was added to the predictive model of in-hospital hypotension composed of traditional risk factors **(**Table 4 and Supplement 4).

**Table 4:** Comparison of the predictive performance of the in-hospital hypotension with and without the delta neutrophil index (DNI).

Prediction model	Incremental value of DNI added to predictive model composed of traditional risk factors									
	AUROC (95% CI)	Difference of AUROC (95% CI)	*P*	NRI (95% CI)	*P*	IDI (95% CI)	*P*			
**(A)**										
Reference (Ref.) model	0.814 (0.750–0.879)									
Ref. model + DNI^a^	0.819 (0.754–0.884)	0.005 (−0.022–0.031)	0.731	0.127 (0–0.254)	0.049*	0.027 (0.004–0.05)	0.023*			
**(B)**										
**Prediction model**	**Comparison between predictive model composed of traditional risk factors and traditional risk factors combined with DNI**									
	**Sensitivity (95% CI)**	***P***	**Specificity (95% CI)**	***P***	**Accuracy (95% CI)**	***P***	**NPV (95% CI)**	***P***	**PPV (95% CI)**	***P***
Ref. model	72.1 (60.9–83.4)	Ref.	81.4 (76.5–86.3)	Ref.	79.5 (75–84.1)	Ref.	49.4 (39.1–59.8)	Ref.	92.1 (88.4–95.7)	Ref.
Ref. model + DNI^a^	63.9 (51.9–76)	0.020	90.1 (86.3–93.8)	<0.001	84.8 (80.8–88.9)	0.001	61.9 (49.9–73.9)	<0.001	90.8 (87.2–94.5)	0.170

*P < 0.05, ^a^Variable was logarithmically transformed before analyses. Abbreviations: AUROC, area under the receiver operating characteristic; CI, confidence interval; DNI, delta neutrophil index; IDI, integrated discrimination improvement; NRI, net reclassification index; PPV, positive predictive value; NPV, negative predictive value; Reclassification tables comparing two prediction models (See Supplement 4). Reference model = qSOFA score + Heart rate + Body temperature + Platelet count + Neutrophil ratio + Prothrombin ratio + Serum albumin level + Serum alanine aminotransferase level + Total bilirubin + C-reactive protein.

### Comparison of 30-day mortality between groups according to treatment modality in patients with higher DNI values

In the present study, the 30-day mortality rates of total patients and those with DNI ≤ 3.3% were 3.9% and 1.6%, respectively. However, that of patients with DNI > 3.3% was 7.6%. On analyzing 30-day mortality according to treatment modality in patients with DNI > 3.3%, we found a mortality rate of 17.4% in patients who received only antibiotics; in contrast, patients who were managed with aggressive treatment, including antibiotic therapy with percutaneous drainage or surgical intervention, had a mortality rate of 5.2%. However, this difference was not significant between the two groups (p = 0.069). In patients with DNI < 3.3%, there was no difference between the two groups (1.3% vs. 1.8%) according to the treatment modality (p > 0.999) (Supplement 5). Clinical characteristics of the patients in our study according to the treatment modality are shown in Supplement 6. Initially stable patients with PLA who received aggressive treatment had a higher cumulative survival rate than those who received conservative management [hazard ratio (HR): 0.277, 95.0% CI: 0.051–0.1.508; P = 0.04] in (Supplement 7).

### External validation of cut-off value

In total, 155 patients were included in the validation cohort during the study period (Supplement 8). Twenty-two patients (14.2%) developed in-hospital hypotension. The 30-day mortality rate was 3.2%. When the cut-off of the study cohort was applied to the validation cohort, the sensitivity and specificity were 81.8% (95% CI, 65.7–97.9) and 54.1% (95% CI, 45.7–62.6), respectively, in predicting in-hospital hypotension. DNI values ≥ 3.3% at ED admission (OR, 5.31; 95% CI: 1.71–16.54; p < 0.001) also remained significantly associated with an increased risk of in-hospital hypotension in the validation cohort.

## Discussion

The present study demonstrated that a high DNI at ED admission was an independent predictor of the development of in-hospital hypotension in initially stable patients with PLA. DNI > 3.3% at ED admission was significantly associated with in-hospital hypotension. The development of in-hospital hypotension in initially stable patients with PLA was closely associated with an increased 30-day mortality. Despite patients having initially stable hemodynamics, the development of shock or in-hospital hypotension in initially stable patients with PLA substantially increases mortality and is associated with serious complications, such as acute kidney injury and acute respiratory distress syndrome^2^.

A previous study^4^ reported that the development of shock in patients with PLA increased mortality up to 22-fold and extended the length of hospitalization, whilst consuming a substantial amount of healthcare resources. In initially stable patients with PLA, our study suggested that the development of in-hospital hypotension was closely associated with increased 30-day mortality. Furthermore, a reliable study^16^ showed that mortality was associated with the duration of hypotension before the commencement of antibiotic treatment. Hence, accurate risk stratification of the development of in-hospital hypotension is a prerequisite for improving patient prognosis through appropriate therapy, including rapid antibiotic administration, urgent abscess drainage, and professional interventions^16^.

Recently, Cho *et al*.^2^ reported older age, malignancy, lower SBP, tachypnea, and increased lactate and procalcitonin to be predictors of the development of shock in initially stable patients with PLA. Although the Cho *et al*.^2^ study identified clinical predictors of shock, which can be determined readily at the bedside, the authors did not suggest optimal cutoff values for several predictors. Therefore, these may be difficult to apply directly to clinical practice. In the present study, the DNI was also found to be an independent predictor of the development of in-hospital hypotension. A DNI ≥ 3.3% at ED admission was associated with a significantly increased risk of developing in-hospital hypotension in initially stable patients with PLA.

To date, there have been relatively few clinical studies of prognostic factors for PLA due to its relatively low prevalence and mortality^3^. Kuo *et al*.^17^ reported that Mortality in Emergency Department Sepsis (MEDS) scores at ED admission are a significant predictor of mortality in initially stable patients with PLA. MEDS scores include clinical signs, such as shock and tachycardia. Therefore, it is unclear whether MEDS scores can predict severity, even before the appearance of clinical symptoms^17^. Acute Physiology and Chronic Health Evaluation II scores have been useful in several studies^5,18^ for predicting the outcome of patients admitted to the intensive care unit (ICU). However, Acute Physiology and Chronic Health Evaluation II scores, originally designed to measure disease severity in patients admitted to the ICU, are based on complex laboratory data. Therefore, at the time of ED admission, its use does not seem feasible^17,19^.

The DNI reflecting the fraction of circulating immature granulocytes in the blood has the additional benefit of being automatically analyzed along with the CBC, which is routinely and rapidly determined in critically ill patients without additional time, cost, and equipment, unlike CRP, lactate, and procalcitonin^15^. Given the simplicity of the measurement and its cost effectiveness, our findings suggest that DNI could be a promising predictor of in-hospital hypotension in initially stable patients with PLA. Previous studies have reported that a high DNI is significantly associated with poor outcomes in patients with specific disease conditions and can predict the severity of infectious diseases. Although we cannot directly compare the different studies, their findings suggest that a high DNI may be positively associated with a higher disease severity. Park *et al*.^13^ demonstrated that a DNI of >6.5% was a promising diagnostic marker of severe sepsis and septic shock in the first 24 hours of admission to an ICU. Similar to our findings, a high DNI was present up to 12 hours before the onset of circulatory or organ failure^13^. Furthermore, Yune *et al*.^15^ reported a DNI of >8.4% on Day 1 (HR: 3.23) and a DNI of >10.5% on Day 2 (HR: 3.29) as being associated with a significant increase in 30-day mortality in patients with out-of-hospital cardiac arrest. The present study showed that DNI ≥ 3.3% at ED admission was significantly associated with in-hospital hypotension (OR: 5.37).

Previous studies^20,21^ have proposed several pathogenic hypotheses to elucidate the mechanism underlying the release of immature granulocytes in the early stages of sepsis. In poorly controlled local infection and systemic microbial infection, neutrophils are in high demand and need to be regenerated in large numbers. Hence, severe systemic infection can switch from steady-state granulopoiesis to emergency granulopoiesis, which is characterized by considerably enhanced de novo generation of neutrophils^22^. DNI may be associated with an increased cellular turnover and a rapid release of mature and immature granulocytes from bone marrow into the peripheral blood^22,23^. Guerin *et al*.^21^ revealed that sepsis was associated with an increased frequency of circulating IGs, which was a good predictive value for sepsis deterioration 48 hours after admission. This finding was in agreement with our data, which showed that DNI reflecting the IG count could be closely associated with the deterioration in patients with PLA.

*Klebsiella pneumoniae* infection has been shown to be an independent predictor of the development of septic shock in initially stable patients with PLA^24^. A previous study by Cho *et al*.^2^ identified *Klebsiella pneumoniae* as the most common pathogen in blood (22.8%) and pus (54.1%) cultures. Our findings are consistent with the results of previous Asian studies. However, the disadvantage that it takes a long time to obtain the results and bacteria cannot be detected in culture may be an obstacle to its clinical applicability for predicting the severity of PLA.

Law *et al*.^25^ evaluated the effectiveness of the WBC, ANC, and CRP level as biomarkers for predicting the treatment outcomes of initially stable patients with PLA. The WBC has a low diagnostic performance for infection due to leukopenia in immunocompromised patients with infection and several coexisting noninfectious factors^25^. Despite its cost effectiveness, the rapid reduction in CRP levels was useful for predicting favorable outcomes and the adequacy of antibiotic therapy in initially stable patients with PLA^25^. A recent study^26^ reported that the NLR was associated with a significantly poorer prognosis in patients with PLA. The present study showed that the DNI at ED admission had similar predictability as qSOFA scores. Moreover, the DNI at ED admission was superior to the WBC, ANC, CRP level, and NLR for predicting the development of in-hospital hypotension. Integrated discrimination improvement (IDI) and net reclassification improvement (NRI) are proposed indicators for identifying improvement of reclassification in a nested model, showing how predictive power improves when DNI is added to traditional risk factors. The addition of DNI yielded a significantly positive IDI and NRI for the prediction of in-hospital hypotension. The present study suggests that DNI can be an ancillary tool to help determine the modality for treatment. Future prospective, multicenter studies with a larger number of patients are needed to validate the usefulness of the DNI for determining the adequacy of treatment and as a marker of appropriate time points for urgent intervention in initially stable patients with PLA.

This study has several limitations. First is its retrospective design based on the inclusion of a patient cohort derived from a single, tertiary, academic hospital. Therefore, there is the potential for selection bias due to the difficulty in controlling for confounding factors. Second, although our hospital endeavored to standardize treatment for PLA, the prognosis may have varied due to the individual administration of antibiotics to patients with different pathogens and the different time points at which intervention or surgery for urgent abscess drainage was required. Third, although the present study demonstrated that development of in-hospital hypotension was associated with increased 30-day mortality, careful attention should be paid while interpreting these results due to the relatively low incidence of PLA mortality (3.9%) in our study. Finally, Pencina *et al*.^27^ suggested that NRI values of <0.2, 0.2–0.6, and >0.6 should be considered weak, intermediate, and strong, respectively. In this study, the NRI value on adding DNI to the model composed of traditional risk factors was approximately 0.127 (95% CI: 0–0.254), showing weak reclassification ability according to the above criteria. Therefore, we should be cautious while clinically applying DNI. However, considering the practicality and cost-effectiveness of DNI, it may still be clinically valuable in patients with PLA, despite the weak NRI observed in the present study.

We found that a high DNI value independently predicts development of in-hospital hypotension within 24 hours of admission in initially stable patients with PLA. DNI is measured at the same time as CBC. It has the advantage of being an easy and rapid measurement. Therefore, the addition of DNI to traditional predictors may serve as a promising ancillary test for rapid risk stratification in initially stable patients with PLA. We suggest that physicians be more attentive toward initially stable patients with PLA showing high DNI values. Future prospective, multicenter studies with larger patient populations are needed to validate the usefulness of DNI as an independent or ancillary predictor, by adding DNI to several other traditional predictors in evaluating initially stable patients with PLA.

## Methods

### Study population

We conducted a retrospective, observational study in the emergency department (ED) of Yonsei University College of Medicine Severance Hospital (Seoul, Republic of Korea), a university-affiliated, tertiary-level referral hospital with an annual census of approximately 85,000 visits. The study was reviewed and approved by the Institutional Review Board of Yonsei University Health System (Seoul, Republic of Korea) (approval no.: 3-2017-0303). The need for written informed consent was waived because of the retrospective study design.

We retrospectively identified 308 consecutive patients (>18 years) who were hemodynamically stable at presentation and diagnosed with PLA who were admitted to the ED between January 1, 2011 and September 30, 2017. The data of patients who were diagnosed with PLA during a single admission to the ED and had a final diagnosis of PLA (International Classification of Diseases, 10^th^ edition code, K75.0) based on diagnostic or therapeutic techniques performed in the ED were retrospectively analyzed. PLA was defined if at least one of the following criteria was present: (1) positive culture of liver aspirate or surgical sample from a discrete liver abscess; (2) diagnostic imaging findings consistent with a liver abscess with a concurrent positive blood culture; and (3) diagnostic imaging findings consistent with a liver abscess with a defined radiological and clinical response to antimicrobial management, even if blood cultures were negative or not performed^2,28^.

The primary endpoint was the development of in-hospital hypotension. To investigate the development of in-hospital hypotension, we excluded patients if they had hypotension (defined as systolic blood pressure [SBP] <90 mmHg, or mean arterial pressure <65 mmHg, or the need for intravenous catecholamine infusion) or an elevated lactate level of ≥4.0 mmol/L at ED admission^2^. Other exclusion criteria included patients referred from other hospitals after commencing antimicrobial therapy, those with amoebic liver abscess or infected biloma, those receiving chemotherapy within 7 days, and those with a history of previous or current hematological malignancy^2,8^.

In-hospital hypotension was defined as sepsis-induced hypotension (SBP <90.0 mmHg, or mean arterial pressure <65.0 mmHg, or a reduction in SBP of >40.0 mmHg from baseline) persisting for >60 minutes without signs of hypovolemia (bleeding, dehydration, vomiting, diarrhea, or adverse drug effects) or cardiac dysfunction 1–24 hours after admission to the ED^2,7,29^. Radiological studies were performed in patients with suspected septic shock in the ED. These patients were treated with empirical broad-spectrum antibiotics immediately after clinical diagnosis^2^. Patients who developed intractable hypotension during hospitalization were treated according to the revised early goal-directed therapy protocol for critical care, including fluid resuscitation and organ support^30^.

### Data collection

The electronic medical records of all patients diagnosed with PLA were reviewed. Data were collected on demographic characteristics (age; sex; prior medical history, including hypertension, diabetes, liver cirrhosis, chronic kidney disease, cardiovascular disease, and malignancy; hemodynamic parameters; laboratory test results; radiological findings, including abscess size; in-hospital course and clinical outcome, including the development of in-hospital hypotension and 30-day mortality; the main type of treatment received; and the period of follow-up). The DNI for each patient was determined using venous blood collected in ethylenediaminetetraacetic-containing vacutainers at ED admission (within 15 minutes of admission to the ED). To assess the DNI, the same type of hematology analyzer (ADVIA 2120; Siemens, Forchheim, Germany) was used as that for the analysis of complete blood counts (CBCs). Quick sequential organ failure assessment (qSOFA) scores were measured at admission to the ED to evaluate the clinical severity of each patient.

### DNI measurements

The specific analyzer performs DNI measurements using two different systems based on a cytochemical myeloperoxidase tungsten-halogen channel and a laser diode channel^8–10^. The cytochemical myeloperoxidase tungsten-halogen channel measures and differentiates neutrophils, eosinophils, lymphocytes, monocytes, and large unstained cells based on size and myeloperoxidase staining intensity^8–10^. The laser diode channel calculates, classifies, and counts cell types according to lobularity/nuclear density and size^8–10,12^. The DNI is then calculated by subtracting the fraction of mature polymorphonuclear neutrophils from the sum of the myeloperoxidase-reactive cells, detecting circulating immature granulocytes as the leukocyte subfraction^11–13^.

### Statistical analyses

Data were assessed for normality using the Kolmogorov-Smirnov test. Demographic and clinical data are presented as the median and interquartile range, mean ± standard deviation, or percentage and frequency, as appropriate. Continuous variables were compared using a 2-sample *t*-test or the Mann-Whitney *U* test. Categorical variables were compared using the Chi-square test or Fisher’s exact test. Because of the skewness of the histogram of the DNI distribution, the DNI was transformed as natural logarithm of DNI value plus 1 before analyses. Multivariable logistic regression analysis was performed to examine the relationship between DNI and the development of in-hospital hypotension. The results of the multivariable logistic regression analysis are presented as odds ratios (ORs) and 95% confidence intervals (CIs).

Areas under the receiver operating characteristic (ROC) curves (AUROCs) were used to assess the ability of the DNI to predict the development of hypotension using ROC curves. The optimal cutoff value for the DNI was determined using Youden’s index. Kaplan-Meier survival curves were also plotted using 30-day mortality data. The hypotension and non-hypotension groups were compared using the log-rank test.

ROC curves were constructed to assess the diagnostic performance of the DNI and other clinical parameters. AUROCs were calculated and compared using the Delong method. Integrated discrimination improvement (IDI) and net reclassification improvement (NRI) were evaluated to analyze the extent to which the addition of the DNI to the reference model improved predictability. To determine 95.0% CIs and *P*-values for IDI and NRI, a standard bootstrap method was used with resampling performed 1,000 times.

To identify significant relationships between treatment modality and 30-day mortality, conservative management using only antibiotics and aggressive treatments, including antibiotics and percutaneous drainage or surgical intervention, were compared using Kaplan-Meier survival curves and the log-rank test.

To validate the cut-off points in this study, the external validation was retrospectively conducted with a validation cohort collected between January 2010 and July 2014 at Yonsei University College of Medicine, Gangnam Severance Hospital (Seoul, Republic of Korea), which attends to 50,000 patients in the ED annually. We collected all data of the validation cohort according to the inclusion and exclusion criteria of this study.

All statistical analyses were conducted using SAS software (version 9.2; SAS Institute Inc., Cary, NC, USA), R software for Windows (version 3.2.5; the R foundation for statistical computing, Vienna, Austria [http://www.R-project.org/]), and MedCalc (version 12.7.0; MedCalc Software, Ostend, Belgium). A *P* < 0.05 was considered statistically significant.

## Declarations

### Ethics approval and consent to participate

The study was approved by the institutional review board (approval no.: 3-2017-0303) of Yonsei University Health System (Seoul, Republic of Korea), which waived the requirement for written informed consent because of the retrospective nature of the study. All methods were performed in accordance with the relevant guidelines and regulations.

## Supplementary information

supplements

## Data Availability

The datasets generated during and/or analysed during the current study are available from the corresponding author on reasonable request.
