# Supplementary material for: The Delta Neutrophil Index Predicts the Development of In-hospital Hypotension in Initially Stable Patients with Pyogenic Liver Abscess
Source: Sci Rep. 2019 Aug 20;9:12105. doi: 10.1038/s41598-019-48588-1 (PMC6702230; doi:10.1038/s41598-019-48588-1)
Supplement: Supplementary file 1 — supplements [file 41598_2019_48588_MOESM1_ESM.docx]

**Supplementary Information**

**The Delta Neutrophil Index Predicts the Development of In-hospital Hypotension in Initially Stable Patients with Pyogenic Liver Abscess**

Taeyoung Kong, MD ^1,†^, Yoo Seok Park, MD, PhD ^1,†^, Hye Sun Lee, PhD ^2^, Sinae Kim, MS^2^, Jong Wook Lee, MD ^3*^, Je Sung You, MD, PhD ^1*^, Hyun Soo Chung, MD, PhD ^1^, Incheol Park, MD, PhD ^1^, and Sung Phil Chung, MD, PhD ^1^

^1^Department of Emergency Medicine, Yonsei University College of Medicine, Seoul, Republic of Korea;

^2^Department of Research Affairs, Biostatistics Collaboration Unit, Yonsei University College of Medicine, Seoul, Republic of Korea;

^3^Department of Laboratory Medicine, Konyang University Hospital, Republic of Korea

^†^These authors contributed equally to this work

***Co-corresponding authors:**

Je Sung You, M.D., Ph.D.

Department of Emergency Medicine, Yonsei University College of Medicine

211 Eonju-Ro, Gangnam-Gu, Seoul 135-720, Republic of Korea

Tel.: +82-2-2019-3030

Fax: +82-2-2019-4820

E-mail: [youjsmd@yuhs.ac](mailto:youjsmd@yuhs.ac)

Jong Wook Lee, M.D.

Department of Laboratory Medicine, Konyang University Hospital

158 Gwanjeodong-Ro, Seo-Gu, Daejeon, Republic of Korea

Tel.: +82-42-600-9279

Fax: +82-42-600-9272

E-mail: [lee423619@kyuh.ac.kr](mailto:lee423619@kyuh.ac.kr)

**Supplement 1.** Distribution of delta neutrophil index (DNI) according to development of in-hospital hypotension.

(A) Raw (untransformed) DNI values, (B) Logarithmically transformed DNI values


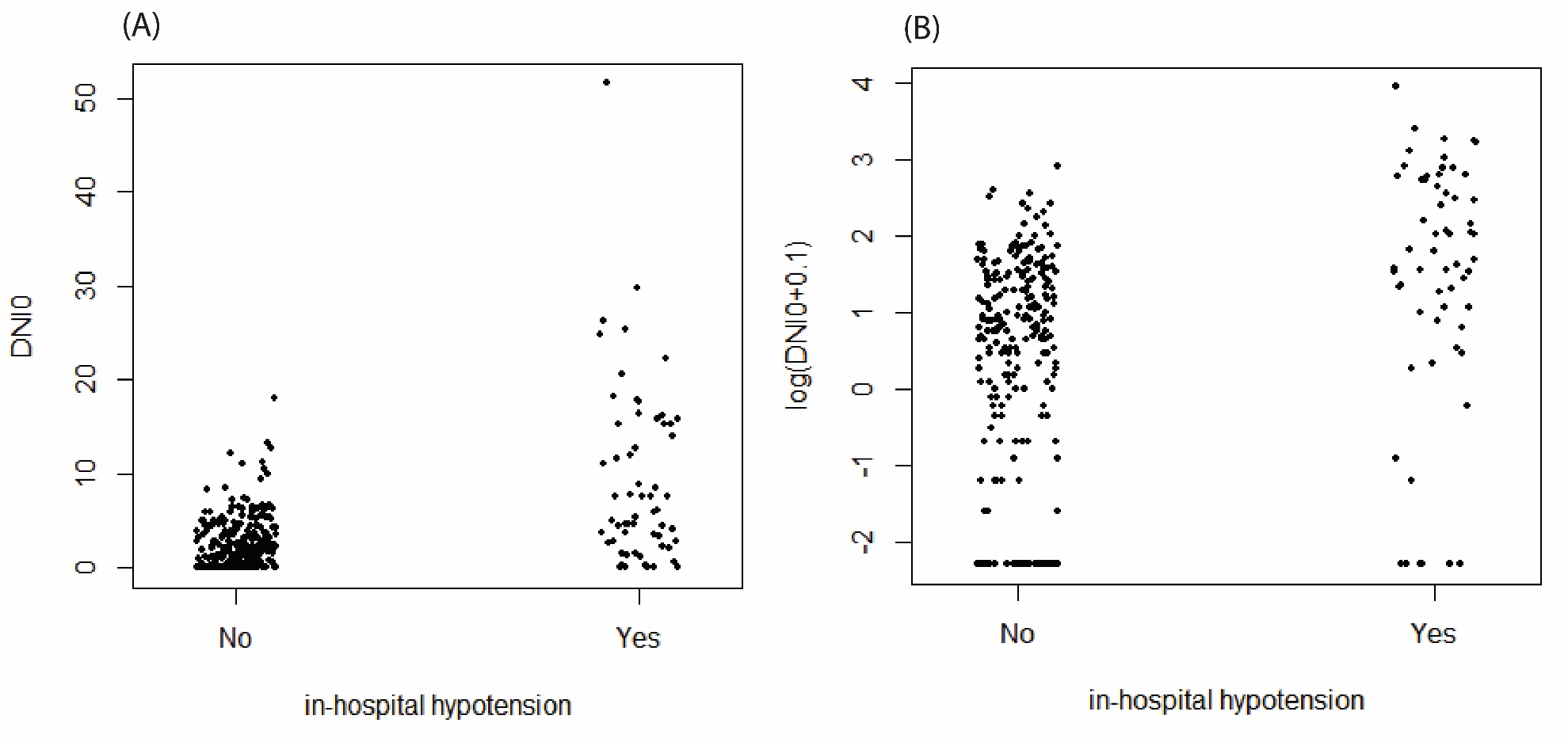


**Supplement 2.** Univariable logistic regression analysis of predictors for the development of in-hospital hypotension.

| **Variables** | **Univariable logistic regression** | |
| --- | --- | --- |
|  | **OR (95% CI)** | ***P*** |
| Age (per 1 year) | 1.021 (0.998-1.045) | 0.078 |
| Male gender (vs Female) | 0.915 (0.515-1.626) | 0.762 |
| qSOFA score (per 1point) | 3.159 (2.011-4.962) | <0.001* |
| **Initial vital sign** |  |  |
| MBP (per 1mmHg) | 0.946 (0.920-0.972) | <0.001* |
| Heart rate (per 1bpm) | 1.038 (1.022-1.055) | <0.001* |
| Body temperature (per 1^o^C) | 1.486 (1.145-1.928) | 0.003* |
| **Radiology features** |  |  |
| Size of abscess (per 1cm) | 0.985 (0.887-1.095) | 0.786 |
| Multiple lesions (vs Single) | 1.140 (0.626-2.078) | 0.668 |
| Bi-lobar involvement (vs Uni) | 1.484 (0.718-3.065) | 0.286 |
| **Comorbidity** |  |  |
| Hypertension | 1.020 (0.575-1.811) | 0.945 |
| Diabetes mellitus | 1.038 (0.555-1.942) | 0.907 |
| Chronic kidney disease | 3.142 (0.684-14.426) | 0.141 |
| Malignancy | 1.527 (0.868-2.686) | 0.142 |
| Liver cirrhosis | 3.396 (0.884-13.050) | 0.075 |
| Cardiovascular disease | 1.326 (0.539-3.264) | 0.539 |
| **Laboratory data** |  |  |
| WBC count (per 10^3/μL) | 0.981 (0.934-1.031) | 0.454 |
| Hemoglobin (per 1g/dL) | 0.928 (0.815-1.058) | 0.263 |
| Platelet count (per 10^3/μL) | 0.994 (0.991-0.997) | <0.001* |
| Neutrophil ratio (per 1%) | 1.057 (1.014-1.102) | 0.009* |
| Prothrombin time (per 1%) | 0.985 (0.968-1.003) | 0.092 |
| Creatinine (per 1mg/dL) | 1.193 (0.963-1.479) | 0.107 |
| Albumin (per 1g/dL) | 0.529 (0.320-0.872) | 0.013* |
| ALT (per 1 IU/L) | 1.005 (1.001-1.008) | 0.009* |
| Total bilirubin (per 1mg/dL) | 1.005 (1.001-1.008) | 0.009* |
| C reactive protein (per 1mg/L) | 1.004 (1.000-1.007) | 0.023* |
| DNI ^a^ (per 1%) | 1.923 (1.476-2.505) | <0.001* |

^*^*P* < 0.05, ^a^Variable was logarithmically transformed before analyses.

Abbreviations: ALT, alanine aminotransferase; CI, confidence interval; DNI, delta neutrophil index; MBP, mean blood pressure; OR, odds ratio; qSOFA, quick sequential organ failure assessment; WBC, white blood cell.

**Supplement 3.** AUROC curve comparison of scoring systems and biomarkers for predicting the development of in-hospital hypotension.

| Variables | AUROC (95% CI) | ***P*** | ***P*** (vs.DNI) | ***P*** (vs.WBC) | ***P***  (vs.Neutrophil ratio) | ***P*** (vs.NL ratio) | ***P*** (vs.qSOFA) | ***P*** (vs.CRP) | ***P*** (vs.ANC) |
| --- | --- | --- | --- | --- | --- | --- | --- | --- | --- |
|  |  |  |  |  |  |  |  |  |  |
| DNI | 0.742 (0.661-0.823) | <0.001* | Ref. | 0.005* | 0.306 | 0.017* | 0.18 | 0.005* | <0.001* |
| WBC count | 0.567 (0.470-0.663) | 0.174 | 0.005* | Ref. | 0.103 | 0.442 | 0.055 | 0.68 | 0.283 |
| Neutrophil ratio | 0.695 (0.613-0.777) | <0.001* | 0.306 | 0.103 | Ref. | <0.001* | 0.709 | 0.064 | <0.001* |
| NL ratio | 0.629 (0.572-0.684) | 0.003* | 0.017* | 0.442 | <0.001* | Ref. | 0.355 | 0.538 | 0.274 |
| qSOFA score | 0.676 (0.604-0.748) | <0.001* | 0.18 | 0.055 | 0.709 | 0.355 | Ref. | 0.121 | <0.001* |
| CRP | 0.595 (0.517-0.673) | 0.017* | 0.005* | 0.68 | 0.064 | 0.538 | 0.121 | Ref. | 0.022* |
| ANC | 0.461 (0.365-0.558) | 0.433 | <0.001* | 0.283 | <0.001* | 0.274 | <0.001* | 0.022* | Ref. |

^*^*P* < 0.05

Abbreviations: ANC, absolute neutrophil count; AUROC, area under the receiver operating characteristic; CI, confidence interval; CRP, C-reactive protein; DNI, delta neutrophil index; NLR, neutrophil-to-lymphocyte ratio; qSOFA, quick sequential organ failure assessment; WBC, white blood cell.

**Supplement 4.** Reclassification tables comparing two prediction models. Net reclassification Improvement (NRI) for development of in-hospital hypotension based on a combination of DNI and traditional risk factors.

| In-hospital hypotension=Yes (n=61) |  | | |
| --- | --- | --- | --- |
| Risk category  (predictive model composed of traditional risk factors) | Risk category with new model  (predictive model composed of traditional risk factors + DNI^a^) | | |
|  | Low risk (<33%) | Intermediate risk (33-66%) | High risk (>66%) |
| Low risk (<33%) | 19 | 5 | 0 |
| Intermediate risk (33-66%) | 3 | 17 | 6 |
| High risk (>66%) | 0 | 1 | 10 |
| In-hospital hypotension=No (n=247) |  | | |
| Risk category  (predictive model composed of traditional risk factors) | Risk category with new model  (predictive model composed of traditional risk factors + DNI^a^) | | |
|  | Low risk (<33%) | Intermediate risk (33-66%) | High risk (>66%) |
| Low risk (<33%) | 210 | 3 | 0 |
| Intermediate risk (33-66%) | 5 | 22 | 0 |
| High risk (>66%) | 0 | 1 | 1 |

Abbreviations: DNI, delta neutrophil index; qSOFA, quick sequential organ failure assessment. ^a^Variable was logarithmically transformed before analyses.

**Supplement 5.** Comparison of 30-day mortality between groups according to treatment modality in patients with pyogenic liver abscesses


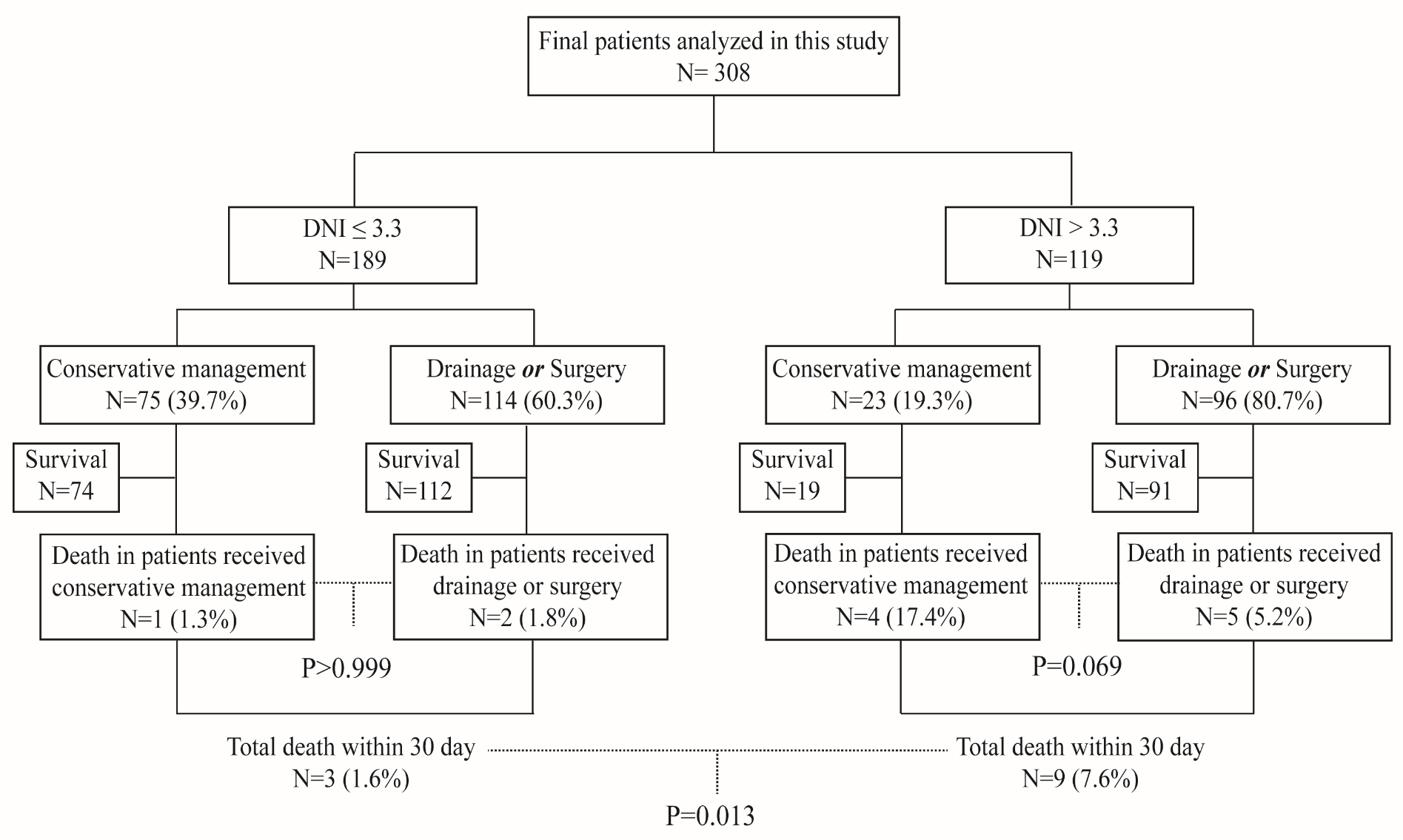


**Supplement 6.** Clinical characteristics of patients according to the treatment modality.

| **Variables** | **Total** | **All patients** | | | **Higher DNI (> 3.3) patients** | | |
| --- | --- | --- | --- | --- | --- | --- | --- |
|  | **N= 308 (100 %)** | **Conservative management** | **Drainage or Surgery** | ***P*** | **Conservative management** | **Drainage or Surgery** | ***P*** |
|  |  | **N=98 (31.8%)** | **N=210 (68.2%)** |  | **N=23 (19.3%)** | **N=96 (80.7%)** |  |
| Age (years) | 63 [55-71] | 64 [57-71] | 62 (54-71] | 0.33 | 66 [58-75] | 62 [55-70] | 0.22 |
| Male gender [n (%)] | 192 (62.34) | 58 (59.18) | 134 (63.81) | 0.44 | 13 (56.52) | 63 (65.63) | 0.41 |
| qSOFA score (point) | 0 [0-1] | 0 [0-1] | 0 [0-1] | 0.14 | 0 [0-1] | 0 [0-1] | 0.69 |
| **Initial vital sign** |  |  |  |  |  |  |  |
| MBP (mmHg) | 85 [77-96] | 86 [78-97] | 85 [76-96] | 0.21 | 83 [74-88] | 80 [74-91] | 0.98 |
| Heart rate (bpm) | 96 [85-108] | 96 [85-11] | 96 [85-106] | 0.87 | 104 [95-118] | 97.5 [87-112] | 0.16 |
| Body temperature (^o^C) | 37.7 [36.9-38.7] | 37.8 [36.7-38.5] | 37.7 [37-38.7] | 0.99 | 38.5 [37.4-38.9] | 37.65 [37-38.8] | 0.12 |
| **Radiology features** |  |  |  |  |  |  |  |
| Size of abscess (cm) | 5 [3.3-6.8] | 3 [2-4.5] | 5.6 [4.3-7.3] | <0.01* | 3 [2.2-4.2] | 5.9 [4.15-7.55] | <0.01* |
| Multiple lesions [n (%)] | 94 (30.52) | 29 (29.59) | 65 (30.95) | 0.81 | 5 (21.74) | 32 (33.33) | 0.28 |
| Bilobar involvement [n (%)] | 47 (15.26) | 9 (9.18) | 38 (18.10) | 0.04* | 3 (13.04) | 22 (22.92) | 0.4 |
| **Timing of treatement** |  |  |  |  |  |  |  |
| Door to antibiotic time (hr) | 3.98 [2.88-5.13] | 4.39 [3.32-5.3] | 3.75 [2.77-4.88] | <0.01* | 4.07 [3.55-5.22] | 3.9 [2.79-4.97] | 0.33 |
| **Comorbidity** [n (%)] |  |  |  |  |  |  |  |
| Hypertension | 120 (38.96) | 33 (33.67) | 87 (41.43) | 0.19 | 4 (17.39) | 41 (42.71) | 0.03* |
| Diabetes mellitus | 84 (27.27) | 29 (29.59) | 55 (26.19) | 0.53 | 4 (17.39) | 23 (23.96) | 0.5 |
| Chronic kidney disease | 7 (2.27) | 4 (4.08) | 3 (1.43) | 0.22 | 1 (4.35) | 2 (2.08) | 0.48 |
| Malignancy | 121 (39.29) | 48 (48.98) | 73 (34.76) | 0.02* | 11 (47.83) | 31 (32.29) | 0.16 |
| Liver cirrhosis | 9 (2.92) | 3 (3.06) | 6 (2.86) | >0.99 | 0 (0.00) | 5 (5.21) | 0.58 |
| Cardiovascular disease | 29 (9.42) | 16 (16.33) | 13 (6.19) | <0.01* | 4 (17.39) | 8 (8.33) | 0.24 |
| **Laboratory data** |  |  |  |  |  |  |  |
| WBC count (10^3/μL) | 11.11 [7.66-15.23] | 9.56 [6.61-14.41] | 12.09 [8.53-15.64] | <0.01* | 11.18 [6.82-16.04] | 11.29 [7.9-16.83] | 0.6 |
| Hemoglobin (g/dL) | 12.15 [10.7-13.4] | 12.1 [10.3-13.2] | 12.2 [10.9-13.6] | 0.3 | 12.6 [10.3-13.9] | 12.4 [11.05-13.85] | 0.95 |
| Platelet count (10^3/μL) | 178 [124-272] | 155.5 [119-252] | 186.5 [134-291] | 0.05* | 148 [124-216] | 148.5 [90-225.5] | 0.82 |
| Neutrophil ratio (%) | 85.4 [79.9-89.5] | 83.7 [76.05-88.65] | 86 [81.2-90] | 0.02* | 90.8 [82.3-93] | 88.6 [84.7-92.3] | 0.63 |
| Prothrombin time (%) | 80 [69-93] | 82 [70-98] | 79 [68-91] | 0.09 | 82 [65-93] | 80 [68.5-94] | 0.81 |
| Creatinine (mg/dL) | 0.85 [0.65-1.08] | 0.78 [0.59-1.07] | 0.88 [0.66-1.11] | 0.02* | 0.84 [0.66-1.12] | 0.99 [0.72-1.32] | 0.22 |
| Albumin (g/dL) | 3.2 [2.8-3.6] | 3.4 [2.9-3.9] | 3.1 [2.7-3.5] | <0.01* | 3.3 [2.8-3.9] | 2.95 [2.6-3.4] | 0.02* |
| ALT (IU/L) | 40 [25-74] | 35 [20-56] | 42 [26-80] | 0.02* | 44 [25-62] | 61 [32.5-96] | 0.07 |
| Total bilirubin (mg/dL) | 1.1 [0.7-1.7] | 1.1 [0.6-1.7] | 1.05 [0.7-1.7] | 0.74 | 1.2 [(0.9-2.3] | 1.3 [0.8-2.05] | 0.51 |
| C reactive protein (mg/L) | 169 [95-234] | 116 [72-191] | 192 [121-250] | <0.01* | 155 [136-208] | 220 [143-276] | 0.02* |
| Lactate (mmol/L) | 1.75 [1.1-2.8] | 1.7 [1.1-3] | 1.8 [1.1-2.8] | 0.9 | 2.55 [1.4-7.9] | 1.95 [1.3-3] | 0.12 |
| Bacteremia (vs None) [n (%)] | 111 (36.04) | 29 (29.59] | 82 (39.05) | 0.11 | 12 (52.17) | 54 (56.25] | 0.41 |
| DNI^a^ | 0.9 [-0.29-1.61] | 0.47 [-2.3-1.19] | 1.08 [0.18-1.72] | <0.01* | 1.69 [1.5-2.73] | 1.8 [1.53-2.17] | 0.89 |

*P<0.05, Data are expressed in median [IQR] and n (%), ^a^Variable was logarithmically transformed before analyses.

Abbreviations: qSOFA, quick sequential organ failure assessment; MBP, mean blood pressure; WBC, white blood cell; AST, aspartate aminotransferase; ALT, alanine aminotransferase; DNI, delta neutrophil index.

**Supplement 7.**

Cumulative survival rates according to treatment modality in pyogenic liver abscess patients with higher DNI values

**
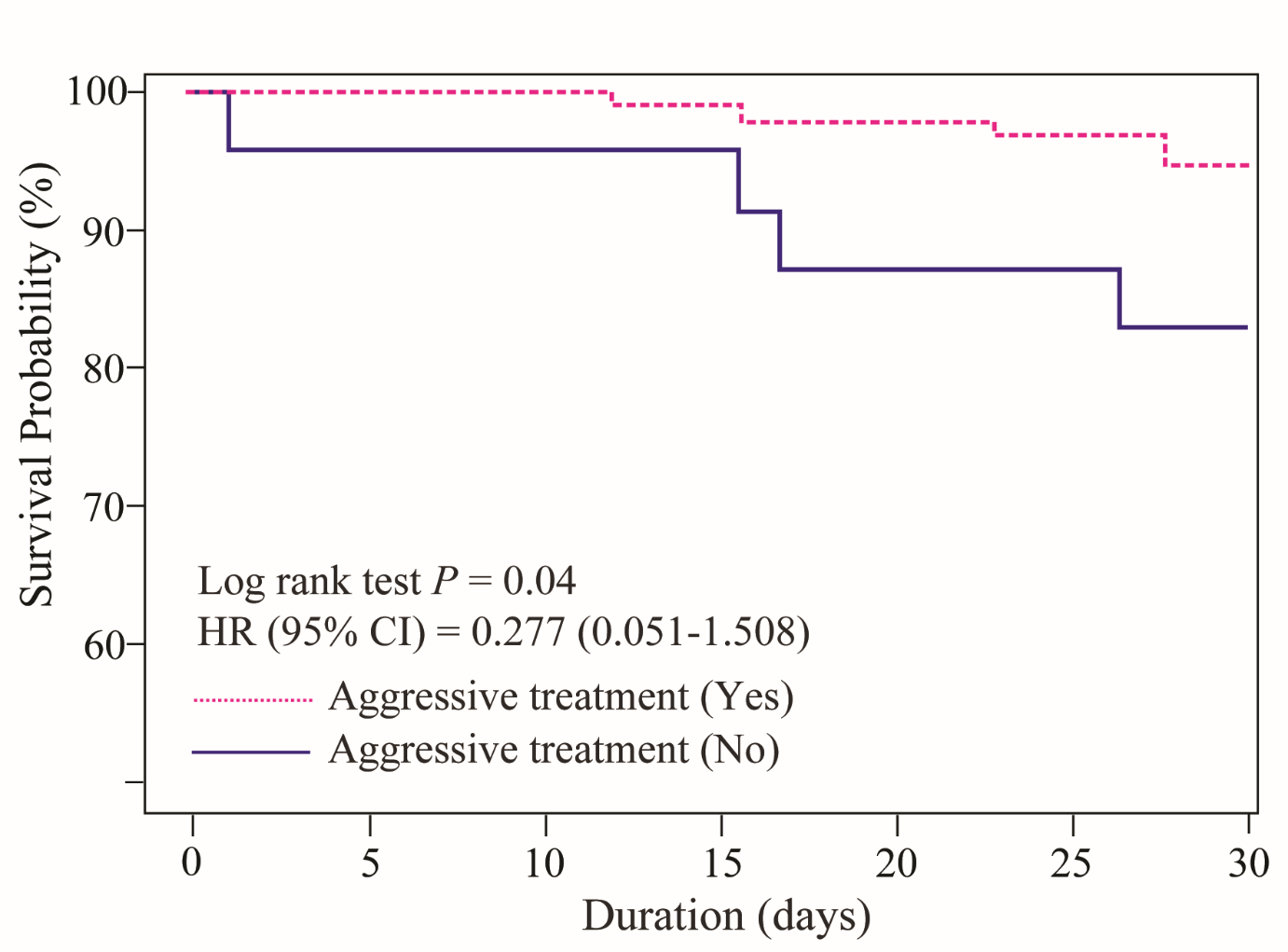
**

**Supplement 8.** Flow chart of patient enrollment in the validation cohort

**
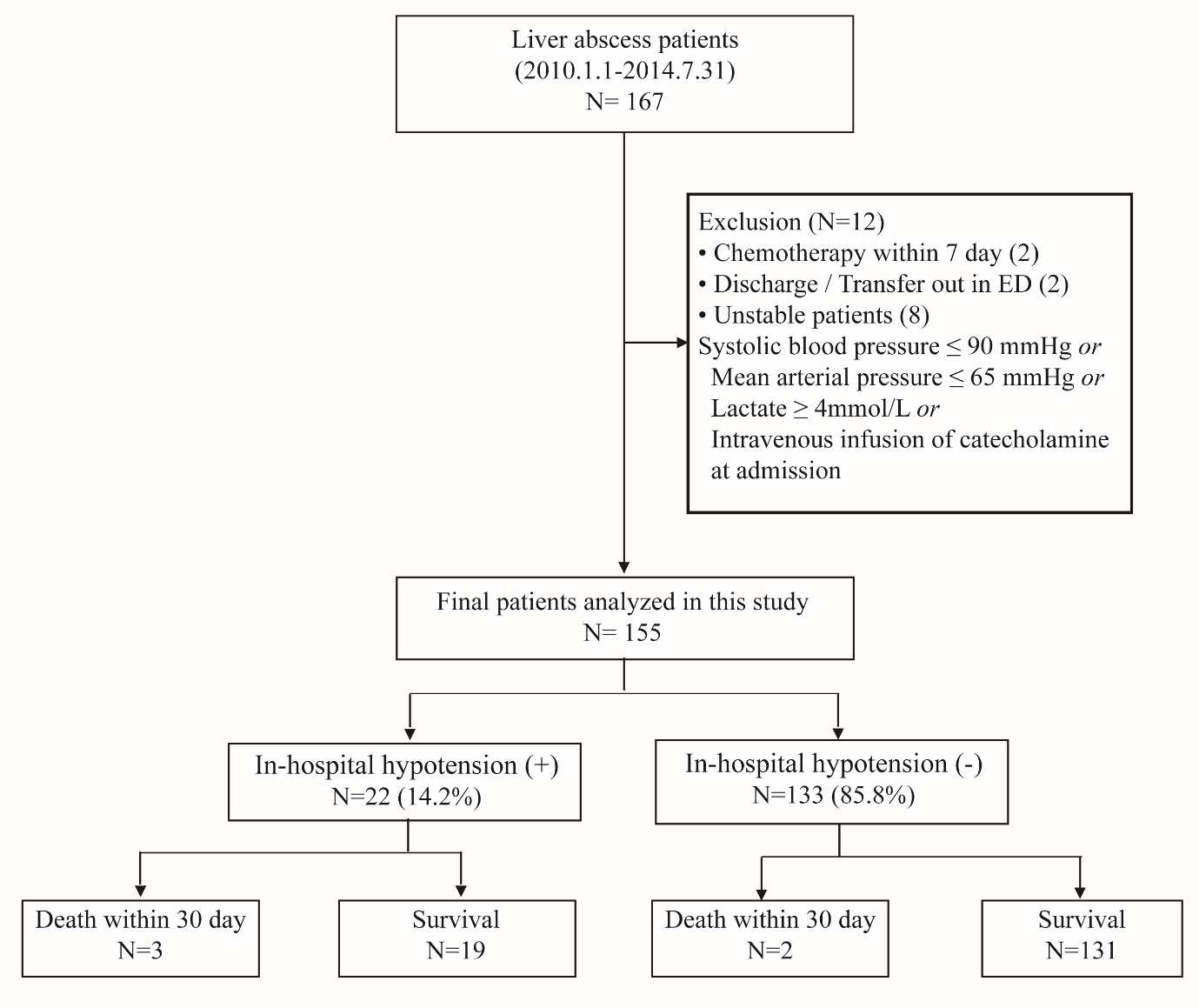
**
